# Mesenchymal Stem Cell Therapy in Traumatic Spinal Cord Injury: A Systematic Review

**DOI:** 10.3390/ijms241411719

**Published:** 2023-07-20

**Authors:** Rodrigo Montoto-Meijide, Rosa Meijide-Faílde, Silvia María Díaz-Prado, Antonio Montoto-Marqués

**Affiliations:** 1 Complejo Hospitalario Universitario de Santiago de Compostela, 15706 Santiago de Compostela, Spain; rodrigo.montoto.meijide@sergas.es; 2 Grupo de Investigación en Terapia Celular y Medicina Regenerativa, Instituto de Investigación Biomédica de A Coruña (INIBIC), Centro Interdisciplinar de Química y Biología (CICA), Universidade da Coruña, 15071 A Coruña, Spain; rmf@udc.es; 3 Departamento de Fisioterapia, Medicina y Ciencias Biomédicas, Universidade da Coruña, 15071 A Coruña, Spain; 4 Unidad de Lesionados Medulares, Instituto de Investigación Biomédica de A Coruña (INIBIC), Complejo Hospitalario Universitario de A Coruña, 15006 A Coruña, Spain

**Keywords:** traumatic spinal cord injury, cell therapy, mesenchymal stem cells

## Abstract

Recovery from a traumatic spinal cord injury (TSCI) is challenging due to the limited regenerative capacity of the central nervous system to restore cells, myelin, and neural connections. Cell therapy, particularly with mesenchymal stem cells (MSCs), holds significant promise for TSCI treatment. This systematic review aims to analyze the efficacy, safety, and therapeutic potential of MSC-based cell therapies in TSCI. A comprehensive search of PUBMED and COCHRANE databases until February 2023 was conducted, combining terms such as “spinal cord injury,” “stem cells,” “stem cell therapy,” “mesenchymal stem cells,” and “traumatic spinal cord injury”. Among the 53 studies initially identified, 22 (21 clinical trials and 1 case series) were included. Findings from these studies consistently demonstrate improvements in AIS (ASIA Impairment Scale) grades, sensory scores, and, to a lesser extent, motor scores. Meta-analyses further support these positive outcomes. MSC-based therapies have shown short- and medium-term safety, as indicated by the absence of significant adverse events within the studied timeframe. However, caution is required when drawing generalized recommendations due to the limited scientific evidence available. Further research is needed to elucidate the long-term safety and clinical implications of these advancements. Although significant progress has been made, particularly with MSC-based therapies, additional studies exploring other potential future therapies such as gene therapies, neurostimulation techniques, and tissue engineering approaches are essential for a comprehensive understanding of the evolving TSCI treatment landscape.

## 1. Introduction

### 1.1. General Overview of Traumatic Spinal Cord Injury

Spinal cord injury (SCI) is a pathological condition that affects the spinal cord and can lead to changes in motor, sensory, and autonomic function [1]. It is a devastating process with major health and socio-economic implications for the patient, their family, and their community.

The annual incidence of traumatic spinal cord injury varies widely worldwide, depending on the demographic characteristics of each country, ranging from 10.4 to 83 cases per million [2]. A 2014 literature review by Lee et al., whose objective was to update the global map of traumatic spinal cord injury and incorporate methods to extrapolate incidence data, reported a global incidence of 23 cases per million population [3]. In a study carried out in Spain analyzing the cases of traumatic spinal cord injuries over a 20-year period, the annual incidence rate was 21.7 cases per million inhabitants [4].

There is a wide variety of possible etiologies for spinal cord injury, such as vascular, congenital, inflammatory, infectious, and, above all, traumatic, which accounts for about 80% of the total, and it is on the latter that we will focus. The most common causes of traumatic spinal cord injury (TSCI) in our setting are falls (54.2%), either high- or ground-level; road traffic accidents (37%); other traumatic causes (5.3%), mainly direct contusions; and sports or recreational accidents (3.5%), mainly diving [4].

The immediate clinical consequences of spinal cord injury include: loss of movement and sensation below the level of injury, flaccid paralysis of the bladder and bowel, and involvement of all body systems below the level of injury (cardiovascular, respiratory, genitourinary, gastrointestinal, musculoskeletal, metabolic, dermatological, and sexual). Similarly, as a consequence of the neurological injury, there is a temporary loss of reflex spinal activity below the level of injury, a phenomenon known as spinal shock, and, as a consequence of the disruption of central sympathetic control, neurogenic shock, which causes bradycardia, hypotension, and hypothermia [5,6].

### 1.2. Pathophysiology of Traumatic Spinal Cord Injury

TSCI occurs when the tissues surrounding and protecting the spinal cord (ligaments, muscles, and bony structures) are unable to absorb the energy generated by the trauma. The spinal cord is injured directly by the trauma itself or indirectly by the displacement of bone or disc fragments [7].

The most common mechanism of TSCI results from a combination of forces that cause violent movements of hyperextension, hyperflexion, and axial compression of the head or trunk, resulting in bone and ligament injuries that ultimately trigger this spinal injury [8]. The magnitude and direction of the traumatic forces determine the type and extent of the bone and ligament injury, with the cervical spine being the most vulnerable due to its high mobility and poor stability. There is also a significant percentage of TSCI that can occur without fracture or dislocation of the spine, such as TSCI in children, due to the greater flexibility of their spine or in adults with significant degenerative changes. On the other hand, complete anatomical transection of the spinal cord occurs in a small percentage of injuries [6].

The mechanism of TSCI consists of two phases: a primary and a secondary phase [7,8,9]. As a result of the initial mechanical trauma, the spinal cord parenchyma is affected, with microhemorrhages that alter blood flow in the central gray matter leading to local infarctions due to hypoxia and ischemia. There is also a loss of nerve conduction in the adjacent white matter. This whole process is known as primary or immediate injury and occurs within the first 0 to 2 h [10].

**Figure 1:**
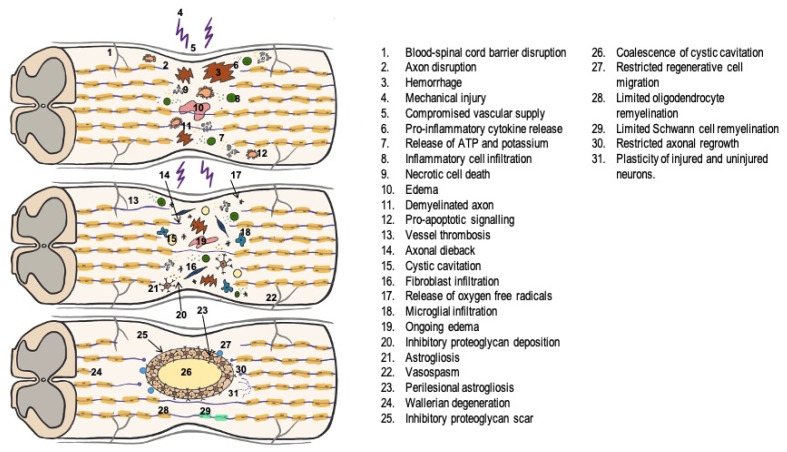
Pathophysiology of traumatic spinal cord injury (modified from Ahuja et al.) [16].

Immediately thereafter, the development of the secondary phase begins. This is the consequence of a series of pathophysiological events triggered by the initial damage, consisting of an inflammatory cascade that destabilizes the axonal membrane, producing an irreversible pattern of spinal cord degeneration and neurolysis with neuronal and glial cell necrosis [7,11]. This secondary phase is further subdivided into an acute, subacute, intermediate, and chronic phase (Figure 1):

1. In the acute phase, lasting between 2 and 48 h, a series of alterations occur, among which the most important are:
  - Release of free radicals from lipid peroxidation of the cell membrane due to the initial trauma [12].
  - Spinal cord ischemia is associated with initial vasospasm, with subsequent reperfusion and the development of edema further exacerbating the injury [13].
  - Ionic alterations such as increased intracellular Ca^2+^ concentration, failure of Na^+^/K^+^ ATPase pumps, activation of voltage-dependent Na^+^ channels, and massive depolarization.

All this, together with a powerful inflammatory response and the release of numerous cytokines (such as tumor necrosis factor-alpha, interleukin 6 and interleukin 1 beta), leads to the apoptosis of neurons, oligodendrocytes, glia cells, and astrocytes [14].

2. During the subacute phase, lasting approximately 2 days to 2 weeks, there is increased perilesional phagocytic activity in an attempt to regenerate the destroyed axons and astrocyte hyperplasia and hypertrophy around the injury, resulting in a glial scar, which acts as a physical and chemical barrier to axon regeneration [15].
3. In the intermediate (2 weeks to 6 months) and chronic phases, this glial scar matures and further extends the injury, preventing axonal regrowth and resulting in the permanent loss of neuronal electrical and functional activity [7,8].

### 1.3. Assessment and Management of Traumatic Spinal Cord Injury

TSCI usually occurs in the context of high-energy trauma, whether it is a traffic accident or falls from great heights, or in less severe injuries, such as in the case of level falls in elderly people or people with diseases affecting the spine [5,7,17].

Most TSCIs occur in polytrauma patients; thus, the initial prehospital care of these patients is performed following the international protocol for Advanced Trauma Life Support, or ATLS, which indicates that the initial management of these patients must be carried out following the ABCDE sequence, which refers to the initials of the following items:

- Airway: maintaining the airway permeable and protecting the spinal cord.
- Breathing: controlling breathing and ventilation.
- Circulation: maintaining a proper hemodynamic status.
- Disability: performing a correct neurological examination.
- Exposure: exposing the whole body for injury assessment and preventing hypothermia by covering the patient [5,18].

The clinical diagnosis of acute spinal cord injury is based on a basic neurological examination. However, in this type of polytrauma patient, said examination may not be possible or be difficult; in addition, many of these patients may be sedated or intubated, which poses additional difficulties for examination. Despite this, there are a number of clinical signs and symptoms that may help suspect a spinal cord injury (Table 1). Therefore, it is very important to take a proper medical history, either through a witness, first responder staff, or patients themselves, when possible [5].

In terms of immobilization at the point of initial care, the current preference is for selective immobilization, trying to identify which patients might benefit from it because the systems used may be associated with complications. These include difficulty accessing the airway or increased intracranial pressure [5,19].

For polytrauma patients, protocols for immobilization have been developed in different guidelines, with the purpose of limiting immobilization to cases where it is really necessary [20]. The most commonly used criteria for out-of-hospital care are the NEXUS (National Emergency X-Radiography Utilization Study) and CCSR (Canadian C-Spine Rule) criteria [21,22]. Both are highly sensitive in terms of excluding significant cervical injuries without a radiographic examination. NEXUS, which sets out 5 criteria for low risk, is easier to use (1. no cervical pain; 2. no focal neurological deficit; 3. normal level of consciousness; 4. lack of intoxication; 5. absence of painful distracting injuries); these criteria, when met, can rule out a cervical spinal cord injury. The CCSR criteria combine high- and low-risk criteria and the ability to rotate the head by 45°. High-risk criteria are age over 65 years, existence of paresthesias, and a dangerous mechanism of injury. Low-risk criteria are a subsequent motor vehicle collision, the patient walking at the scene of the accident, the onset of pain late after the accident, and no spinal pain. Patients are risk-free and, therefore, will not require immobilization if they meet low-risk criteria and are also able to voluntarily rotate their heads by 45° [22].

Immobilization is usually completed using a spinal board to fix the head and a cervical collar [19], although the methods used for immobilization during rescue and transport may differ and should be adapted to the clinical situation of the patient, bearing in mind that the priority is ABC [5].

The ASIA/ISCOS International Standards for the Neurological Classification of Spinal Cord Injury (ISNCSCI) are used for the neurological assessment of TSCI (American Spinal Injury Association/International Spinal Cord Society) [23]. This rating system was developed in 1983 by an international consensus of experts and is reviewed periodically, most recently in 2019 [23]. The new 2019 edition introduced two main concepts, consisting of a new taxonomy for the documentation of non-SCI-related conditions and a revised definition of the applicability of the zone of partial preservation [24].

This internationally recognized assessment system describes the level and extent of SCI based on motor and sensory examination. 

The sensory examination is completed through the testing of 28 dermatomes on the right and left sides of the body (C2 to S4–S5). In each dermatome, light touch (rubbing cotton) and pin-prick sensation are assessed (needle prick) according to a 3-point scale (0 = absent, 1 = deterioration, 2 = normal). In addition, the anal sensation is valued deeply through rectal examination, registering it as present or absent.

The motor examination is performed through the assessment of five key muscles in the upper limbs and another five key muscles in the lower limbs corresponding to paired myotomes (C5: elbow flexors; C6: wrist extensors; C7: elbow extensors; C8: flinger flexors; T1: small finger abductors; L2: hip flexors; L3: knee extensors; L4: ankle dorsiflexors; L5: long toe extensors; S: ankle plantar flexors). The strength of each key muscle is graded on a six-point scale (0 = total paralysis; 1 = palpable or visible contraction; 2 = active movement full range of motion with gravity eliminated; 3 = active movement full range of motion against gravity; 4 = active movement full range of motion against moderate resistance; 5 = normal movement). In addition, the voluntary anal contraction should be tested with the examiner’s finger inserted into the rectum and graded as present or absent.

In addition to the neurological diagnosis, it classifies the injury into different grades. The following order is recommended to determine the classification of spinal cord injury: 1. determine sensory levels for both sides of the body; 2. determine motor levels for both sides of the body; 3. determine the neurological level of injury; 4. determine whether the injury is complete or incomplete; 5. determine ASIA Impairment Scale (AIS) grade; 6. determine the zone of partial preservation. The sensory level is defined as the most caudal intact dermatome for both pin-prick and light touch sensations. The motor level is defined by the lowest key muscle having a grade of at least 3, with key muscles represented by segments above that level graded as 5. The neurological level of injury is the most cephalad of the sensory and motor levels determined in steps 1 and 2. The zone of partial preservation refers to those dermatomes and myotomes below the sensory and motor levels that remain partially innervated [23].

This classification also makes it possible to establish the prognosis of the SCI according to the degree of injury in the first few hours of its development. As the circumstances and reliability of the neurological examination can influence conversion rates for injuries, SCI management guidelines recommend a 72-h scan as the most reliable method for long-term prognosis [25].

### 1.4. Outcomes after Traumatic Spinal Cord Injury

The main predictive factor for recovery in TSCI is the degree of extension of the injury according to the ASIA classification. This classification describes the level and extent of the injury, classifying spinal cord injuries into five degrees (Table 2) [23].

The best time to perform a neurological examination to determine the long-term outcome of the injury is 72 h after the injury, as mentioned in the previous section [25].

According to the guidelines of the Consortium for Spinal Cord Medicine, using the ASIA neurological assessment at the first week of the injury, 80–90% of grade A injuries will remain complete; of those that become incomplete injuries, only 3–6% will recover functionality. About 50% of the patients with grade B injuries will have functionality, taking into account that those with grade B injuries who have preserved painful sensitivity will show a better progression. Approximately 75% of those with grade C injuries will regain ambulation in the community, and among those initially assessed as grade D, functionality is close to 100%, depending on factors such as age, previous co-morbidities, etc. [26].

Although more recent studies, such as the systematic review by El Tecle et al. [27], which analyzed randomized trials, have reported a highly variable conversion of AIS grade A between 0% and 44%, the general percentage of improvement for grade A injuries is between 15–20% [28].

Regarding the factors associated with improvements in AIS grade, aspects such as the level of injury have been pointed out, and a systematic review by Wilson JR reported that patients with complete thoracic injuries have a lower potential for neurological recovery [29]. Other authors have also pointed out the influence of associated injuries, whose presence is related to the greater violence of the trauma and the greater severity of the spinal cord injury. Although some authors have pointed to the contribution of certain demographic factors, such as age and sex, their influence on AIS grade conversion has not been definitively demonstrated. Thus, it has been described that patients under 65 years of age could show greater recovery in motor scores than those over 65 years of age. On the other hand, women have been described as showing a greater potential for recovery of motor score after one year of evolution; however, none of the studies mentioned improvements in AIS grade [30].

### 1.5. Available Therapies

Adequate management of TSCI begins with a suspicion or diagnosis at the point of care, along with transferring the patient to a hospital center. In cases of life-threatening conditions, the patient shall be transferred to the nearest hospital and, as soon as possible, to a referral center for specialized TSCI care [5].

In a hospital setting, the previous life support and immobilization measures must continue while pertinent diagnostic studies are carried out and specific therapeutic measures are initiated [31].

Radiological examination will vary depending on the need to diagnose injuries associated with TSCI. As the presence of vertebral fractures at different levels is common, a radiological examination of the entire spine is generally recommended. The radiological examination of choice is computed tomography (CT) with sagittal and coronal reconstructions, as it is more sensitive and specific than plain radiography and allows for easier identification of fractures and full visualization of the spine in difficult-to-explore areas such as the occipitocervical and cervicothoracic junctions. It also provides a good assessment of spinal canal invasion and allows for the planning of surgical treatment [32].

Magnetic resonance imaging (MRI) is the test that allows for the correct classification of the type of TSCI (edema, hemorrhage, etc.). Together with clinical examination, MRI allows for the prognostic assessment of spinal cord injury. It also allows for the detection of soft tissue and ligament injuries, which can be important in determining the stability of the spine. Therefore, if the patient’s clinical situation allows for it, it should be performed as soon as possible. Sometimes it must be performed urgently, for example, in cases of clinical-radiological discordance or when there is a neurological worsening that may be caused by an epidural hematoma or another cause that requires urgent surgical treatment [5]. According to the AOSPINE Clinical Practice Guidelines, an MRI should be performed in the acute phase to support the neurological outcome; in addition, this MRI serves to support better clinical decision-making [29].

There are currently several therapeutic strategies, some of which are being used in clinical practice while others are being applied at an experimental level (some of which will be analyzed in this systematic review). Clinically, neuroprotection (reduction of secondary damage) and rehabilitation are the cornerstones of TSCI treatment; both are the therapeutic strategies available to reduce the disability caused by the TSCI [33].

Neuroprotection includes pharmacological agents that suppress the immune system or key inflammatory pathways. They were the first major strategies applied to patients, including non-steroidal anti-inflammatory drugs (NSAIDs), cyclosporine, minocycline, and corticosteroids (methylprednisolone, etc.).

Corticosteroids were considered standard treatment in the acute phase of TSCI until 2013. Methylprednisolone is thought to work by reducing cell membrane peroxidation and inflammation, as well as having an immunomodulatory effect by inhibiting neutrophil and macrophage infiltration into the spinal cord. The use of steroids in the treatment of TSCI is not currently recommended to improve the neurological prognosis. There is no evidence to support the clinical benefit of methylprednisolone in the treatment of acute spinal cord injury. However, there is some evidence that high doses of steroids are associated with harmful side effects in terms of respiratory infections and gastrointestinal bleeding, even leading to death. In those injuries with neurological deterioration, the use of short-course corticosteroids could be considered, always weighing the risk-benefit ratio against the side effects [25,34].

Spinal cord perfusion pressure is related to mean arterial pressure and intraspinal pressure, such that spinal cord perfusion pressure is equal to the difference between mean arterial pressure and intraspinal pressure. Therefore, blood pressure management during the acute period after SCI is considered extremely important as a neuroprotective factor since prolonged hypotension can worsen spinal cord ischemia and secondary damage [9]. The current guidelines of the American Association of Neurological Surgeons (AANS) provide Level III recommendations for continuous hemodynamic monitoring and interventions to maintain mean arterial pressure (MAP) above 85 mmHg for the first 7 days after injury. This recommendation is essentially based on two prospective interventional studies without a control group [35].

Current surgical treatment of TSCI involves decompression and stabilization, although there is no agreement as to when this should be carried out: early or late. The main goal of early surgery in acute TSCI is to reduce spinal cord compression and ischemia to optimize the local environment for neurological recovery. Some recent publications, such as the AANS and AOSpine guidelines, support the effectiveness of early surgery [36]. However, a recent systematic review found that there is only limited evidence to support the clinically significant benefit of early decompressive surgery. Surgery is thought to be safe, and while early surgical intervention is increasingly becoming the norm, the evidence supporting its effectiveness is still limited [37].

So far, the mainstay of treating TSCI, based on reducing secondary injury, has failed to produce satisfactory results. However, thanks to major advances in molecular and regenerative medicine over the last two decades, a hopeful future has opened up thanks to the discovery of new therapies based on neuroprotection (such as riluzole, minocycline, G-CSF, FGF-2, and polyethylene glycol), neuroregeneration (chondroitinase ABC, self-assembling peptides, and Rho inhibition), the use of bioactive materials, and especially stem cells [5].

### 1.6. Cell Therapies

Recovery from TSCI is extremely difficult due to the central nervous system’s (CNS) poor ability to regenerate cell and myelin loss and restore neuronal connections. The CNS has poor regenerative abilities due to: (1) the complexity of interconnected neural networks: unlike some other tissues where cells can simply divide and replace damaged or lost cells, when a CNS injury occurs, connections are disrupted, and the complexity of neural networks constitutes a significant obstacle to regeneration; (2) inhibitory environment: the CNS has a complex matrix that contains inhibitory molecules that restrict the growth of neurons and axons, limiting the potential for regeneration; (3) glial scar formation: after CNS injury, astrocytes form a glial scar tissue that contains inhibitory molecules that restrict the growth of neurons and axons; (4) a limited population of neural stem cells; and (5) immune response, which leads to inflammation and, while being part of the healing process, can also have negative effects on neural tissue [38].

Nowadays, cell therapy is considered the most promising treatment for spinal cord injury. The most extensively studied cell types that have shown the best results and the greatest potential are glial cells and stem cells.

Glial cells were the first cells to be used in the treatment of TSCI. They are the most numerous cells in the nervous system, and their main function is to provide support and nutrition to neurons. These cells are responsible for the formation of neuronal networks as well as various metabolic functions and the maintenance of homeostatic balance. There are several types of glial cells, but the most common are the following:

#### 1.6.1. Glial Cells

- Schwann cells

Schwann cells are found in the peripheral nervous system, covering the axons of neurons. Their main function is to synthesize myelin, which enables the conduction of nerve impulses through peripheral nerves. Several studies have demonstrated the efficacy of these cells in patients with TSCI. The main benefits of these cells include neuroprotection, support for axonal regeneration and remyelination, and reduction of glial scarring. In addition, clinical trials in TSCI patients have shown improvement in motor and sensory functions [7,28,39,40,41].

- Astrocytes:

Astrocytes are the most abundant glial cells. They present receptors for several neurotransmitters, such as glutamate or GABA (gamma-aminobutyric acid). They are also essential for the trophic support of neurons and the maintenance of synapses. Their use in TSCI is highly controversial because they are the main agents responsible for the formation of the glial scar, which acts as a physical and chemical barrier to axonal regeneration [42,43]. However, several studies on animals have suggested their ability to prolong neuronal viability [8,15]; therefore, additional studies are needed to clarify their potential benefits.

- Oligodendrocytes:

Oligodendrocytes are the equivalent of Schwann cells in the CNS and are responsible for synthesizing the myelin that surrounds white matter axons. For this reason, they have been used in TSCI therapy for several years due to their ability to remyelinate axons, secrete trophic factors, and reduce glial scarring. A clinical trial conducted in 2019 demonstrated an improvement of at least one grade in the AIS of the patients in this study [16].

- Olfactory Ensheathing Cells:

Olfactory Ensheathing Cells are a specialized subtype of glial cells that surround olfactory neurons (similar to Schwann cells in the peripheral nervous system) and play a key role in axonal regeneration. They are found in the nasal mucosa and olfactory bulb and also secrete neurotrophic factors that promote proper neuronal function. Further studies are needed to confirm their true therapeutic potential, as some studies have revealed improvement in sensitivity in TSCI patients with AIS grade A, but the available data on long-term safety and efficacy are scarce [16].

#### 1.6.2. Stem Cells

In recent years, most efforts and hopes have been placed on stem cell therapy. Stem cells are living cells found in almost all multicellular organisms. These cells have the ability to divide asymmetrically to give rise to other cells similar to themselves, a property known as self-renewal, as well as to divide and differentiate into different specific cell types [44].

Based on their origin, stem cells are commonly classified as adult or somatic stem cells and embryonic stem cells. On the other hand, they can also be classified according to their potency, which is defined as the ability to give rise to different cell types through a process known as cell differentiation. We can distinguish between totipotent (the only ones capable of generating a complete organism), pluripotent, multipotent, oligopotent, and unipotent stem cells. Regarding their capacity to differentiate into different cell types, stem cells can be divided into: (1) totipotent cells: they have the highest level of potency or capacity for differentiation since they can give rise to an entire organism; (2) pluripotent cells: they have the ability to differentiate into any cell type in the body, excluding the placental tissues; (3) multipotent cells (also known as oligopotent cells): they have a more limited but still significant capacity for differentiation into a range of closely related cell types within a specific tissue or organ; and (4) unipotent cells: they have the lowest level of capacity for differentiation since they can only give rise to one specific cell type [45].

**Figure 2:**
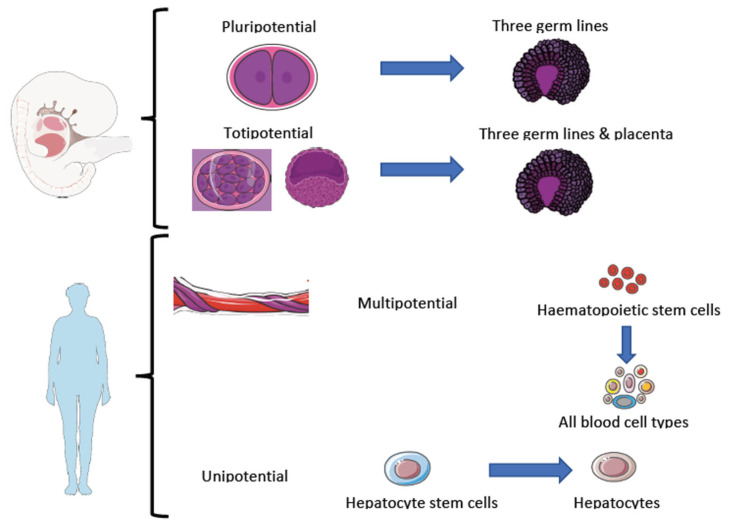
Types of stem cells according to their origin and potency, modified from Mata-Miranda et al. [46]. The figure was partly created using Servier Medical Art, provided by Servier and licensed under a Creative Commons Attribution 3.0 Unported licence.

Thus, the main difference between embryonic and somatic, or adult, stem cells lies mainly in their potency, since embryonic stem cells can be totipotent or pluripotent, whereas adult or somatic stem cells can be multipotent, oligopotent, or unipotent (Figure 2). In addition, pluripotent cells can be isolated from extra-fetal tissues such as amniotic fluid, chorion, amnion, and umbilical cord [46].

The development of multicellular organisms begins with a single cell, the zygote, which divides in a regulated and rapid manner during gestation. The cells from the first two divisions are totipotent stem cells because they are capable of giving rise to both embryonic and extraembryonic tissues (placenta). As these cells divide, their ability to give rise to extraembryonic tissues disappears, and they become pluripotent stem cells, i.e., capable of giving rise to cells of the three germ layers: endoderm, ectoderm, and mesoderm. These are cells that can give rise to all the tissues and organs of the body. At the end of embryonic development, as these cells differentiate into other lineages, their potency decreases, giving rise to multipotent, oligopotent, and finally unipotent cells [47].

Unipotent stem cells, which can give rise to only one type of cell, differ from non-stem cells in that they have the ability to self-renew. Adult stem cells can also give rise to cells of other lineages, depending on the microenvironment into which they are transplanted. This cellular phenomenon, called stem cell plasticity, is often used to treat many diseases, including spinal cord injury [48].

For many years, and in line with the “epigenetic landscape” proposed by Conrad Waddington [49], cell differentiation was understood as a process that occurred in one direction, from an immature state towards a more mature differentiated state. It was also thought that unnecessary genetic information was permanently removed or inactivated in cells that were fully differentiated or committed to a specific differentiation state [50].

However, in 2006, Dr. Shinya Yamanaka et al. made an important discovery in this field. By introducing pluripotency genes into somatic cells, they developed induced pluripotent stem cells (iPSCs). They obtained adult cells with the potential to grow and differentiate similar to embryonic stem cells. This discovery won him the 2012 Nobel Prize in Medicine [51]. The range of possibilities that these induced pluripotent cells have opened up in cell therapy is very broad. However, the most widely used types of stem cells, of which mesenchymal stem cells (MSCs) are the most prominent, are as follows:

- Neural progenitor cells:

These are the only adult stem cells capable of differentiating into the three neural cell lineages (astrocytes, oligodendrocytes, and neurons). They are located in the subventricular zone of the lateral ventricles and the subgranular zone of the dentate gyrus of the hippocampus [52]. Transplantation of these cells into the injured area of spinal cord tissue allows for the recovery of body function by modulating the contribution of astrocytes to the glial scar, promoting neuronal and oligodendrocyte differentiation, and restoring the missing neurons. On the other hand, neural progenitor cells secrete a large number of growth-promoting factors (such as GDN-F, NGF, IGF-1, etc.) that promote the growth and survival of damaged neurons. In addition, the therapeutic effects of these progenitor cells include immunomodulatory effects, enhanced myelination, and improved motor and sensory functions [53]. It is worth noting the study by Levi A. et al., which showed an improvement at the motor level in patients with cervical TSCI, although the study was interrupted for reasons outside the research [54].

- Embryonic cells:

These are totipotent or pluripotent cells. Due to their nature, they have been considered a very interesting and attractive option for the treatment of TSCI. However, studies on animal models have shown that they can contribute to the formation of teratocarcinomas. On the other hand, as these cells are obtained from living human embryos, their use poses an ethical conflict worldwide [55]. For this reason, other therapeutic options are being sought, such as the development of iPSCs.

- Hematopoietic cells:

They are multipotent cells derived from hemangioblasts in the bone marrow. Several studies have demonstrated the safety of these therapies and reported improvements at the motor and sensory levels, although functional neurological recovery in patients has not been demonstrated [56,57].

- Mesenchymal cells:

They are multipotent cells that can regenerate and differentiate into mesoderm-derived cell types, including myocytes, chondrocytes, osteoblasts, and adipocytes. MSCs isolated from different tissues display different cell surface markers that can be used for a range of therapeutic options. They currently constitute the main line of research and have shown the best results in cell therapy for the treatment of TSCI, mainly due to their differentiation and neuronal regenerative capacity. Stem cells possess (1) antiapoptotic properties: since they can secrete various factors and molecules that promote cell survival and prevent cell death, they have the ability to inhibit programmed cell death; (2) anti-inflammatory properties: since they can secrete anti-inflammatory cytokines and other molecules that inhibit the activation and proliferation of immune cells, they regulate the immune response and (3) angiogenic properties: since they can release proangiogenic factors that stimulate endotelial cell proliferation, they promote the formation of new blood vessels [58,59]. There are several reasons that justify the use of MSCs in stem cells to treat TSCI. They are easy to isolate, maintain viability after cryopreservation, have a high multilineage differentiation potential and show little or no immunogenicity after allogeneic transplantation. In addition, they have homing properties, as they are able to migrate to the site of injury [60]. A disadvantage is that their isolation requires patients to undergo bone marrow aspiration under local anesthesia. They can be isolated from bone marrow, adipose tissue, cord blood, and placenta, but with notable therapeutic differences between them. Thus, the most widely used and those that have shown the greatest benefit in TSCI have been those derived from bone marrow, the umbilical cord, and adipose tissue [61]. These cells include:

- Bone marrow mesenchymal stem cells.

These are adult progenitor cells located in bone marrow cavities, around the trabecular bone surface, and in bone marrow sinusoids [62]. These cells are involved in bone repair, regeneration, and hematopoiesis. They can be easily isolated from bone marrow aspirates and easily transplanted into patients, serving as an excellent cell source for clinical transplantation. The introduction of these cells into the site of injury promotes, among other things, axonal regeneration, reduction of the inflammatory response, and glial scarring. According to some authors, transplantation of these cells showed improvements in ASIA and neurological function in patients [63]. In addition, these cells have shown the ability to enhance the effects of Schwann cells when transplanted together by reducing cell apoptosis [64]. However, there have been conflicting clinical results using this type of cell. Several questions remain unanswered regarding the use of bone marrow mesenchymal stem cells for TSCI. These include: (1) determining the best dosage and timing; (2) determining the potential risks (tumorigenicity and immunogenicity) of mesenchymal stem cell therapy; (3) understanding the exact mechanism of action by which these cells exert their therapeutic effects; (4) knowing the survival and persistence of these cells in the injury; (5) determining how MSCs differentiate into neural cells and how they integrate into the injury site; (6) knowing how these cells promote functional recovery and regeneration; (7) studying the interaction between transplanted MSCs and the host immune system; (8) addressing the lack standardized protocols for MSC isolation, expansion and transplantation, which makes it difficult to compare results across different studies; (9) identifying the most suitable candidates for this MSC therapy and determining the factors that influence patient response and (10) knowing the efficacy of these BM-MSCs in combination with other stem cell types or therapies for TSCI. Therefore, it is crucial to perform studies or clinical trials in order to be able to answer or address all these unanswered questions.

- Umbilical cord mesenchymal stem cells:

They are obtained from umbilical cord blood, specifically from the perivascular regions and the umbilical vein. These cells have effects similar to those of bone marrow cells, such as reducing apoptosis or glial scarring. Their potential benefits at the neuronal level have been shown to be greater than those of bone marrow cells, as has a reduction in spinal cord ischemia [65]. On the other hand, clinical trials in patients have shown improvements in motor function and bladder function compared with patients who received rehabilitation alone [63]. The timing of umbilical cord mesenchymal stem cell transplantation can have a significant impact on the effectiveness of therapy for several reasons: (1) disease progression or conditions, which can affect the success of transplantation; (2) pre-transplant treatments, which should not compromise the success of the transplant or interact negatively with the recipient’s body; (3) donor availability, as transplantation relies on the availability of suitable organs or tissues from donors; (4) immune response, as transplantation is typically accompanied by immunosuppressive therapy to prevent rejection; and (5) recovery and rehabilitation, as the recipient needs time to recover from the procedure and undergo rehabilitation.

- Adipose-derived mesenchymal stem cells:

They have a very similar morphology to that of umbilical cord cells but differ in their proliferative capacity and greater availability since adipose tissue contains a higher proportion of stem cells [66]. They also promote tissue and neuronal regeneration and angiogenesis and secrete anti-inflammatory factors [57].

Numerous studies in acute and chronic TSCI have shown that the use of these cells improves the sensitivity of patients but does not reduce the size of the lesion as visible on MRI. Numerous studies on acute and chronic TSCI have shown that the use of adipose-derived mesenchymal stem cells improves the sensitivity of patients but does not reduce the size of the lesion as visible on MRI. This may be due to the action mechanisms of adipose-derived mesenchymal stem cells. These cells have shown the ability to enhance tissue repair and promote regeneration through multiple mechanisms. These include the secretion of various growth factors, modulation of the immune response, and differentiation into specialized cell types. While these mechanisms can lead to functional improvements and increased patient sensitivity, they may not always directly result in a reduction in lesion size as visible on MRI. The effects of these cells on lesion size may be more complex and multifaceted, involving tissue remodeling and regeneration rather than a simple reduction in size.

Several studies and clinical trials have demonstrated the efficacy of MSCs in the treatment of TSCI due to their ability to regulate the immune response [7]. Due to their capacity for neuronal differentiation and self-renewal, these cells allow for the reestablishment of synapses and neuronal circuits. On the other hand, MSCs have also been shown to promote the release of neurotrophic factors that allow for the development of a favorable microenvironment for neuronal repair and axon remyelination [59,60]. However, the mechanism by which MSC therapy promotes functional recovery in TSCI is not well understood [61]. In this manuscript, a systematic review of the scientific evidence for MSC therapy in TSCI was conducted.

### 1.7. Objectives

#### 1.7.1. Main Objective

The main objective was to conduct a systematic review of the clinical evidence available in the scientific literature on the clinical efficacy, safety, and potential role of mesenchymal stem cell therapies in the treatment of people with traumatic spinal cord injuries.

To contribute to the main objective of the study, a number of specific objectives related to the clinical efficacy of mesenchymal stem cell therapy were developed, such as changes in AIS grade and ASIA scores, changes in clinical tests such as urodynamic and neurophysiological studies, and changes in neuroimaging. Furthermore, to assess the safety of this therapy, adverse effects were analyzed.

#### 1.7.2. Specific Objectives

- To analyze changes in AIS (ASIA Impairment Scale) grade.
- To study changes in ASIA sensory and motor scores.
- To evaluate changes in neurophysiological and urodynamic parameters.
- To identify changes in neuroimaging tests.
- To test for the existence of adverse effects of MSC therapy.

## 2. Methods

### 2.1. Search Strategy

Studies were identified by searching electronic databases PUBMED and COCHRANE Library using the following terms and all their possible endings, using the truncation operator “*”: “spinal cord injury”, “stem cells”, “stem cell therapy”, “mesenchymal stem cells” and “traumatic spinal cord injury”. These terms were combined using the boolean operators “AND” and “OR”. The truncation operator “*” was used to locate the largest number of articles possible, as some of them could otherwise be missed due to not using the exact ending of the words. For a more targeted search, the option to search these terms in article titles was used, and Medical Subject Headings (MeSH) terms “Spinal Cord Injuries/therapy” and “Mesenchymal Stem Cells” were used as additional filters.

The following criteria were used to select the studies:

### 2.2. Eligibility Criteria

Inclusion criteria:

- Type of study: randomized and non-randomized clinical trials, cohort studies, case-control studies, and case series.
- Papers are available in English and Spanish until February 2023.
- Human studies.
- Traumatic spinal cord injury at any level and any AIS grade.
- Intervention: MSC therapy.
- Results: improvement in ASIA sensory and motor scores and grade on the AIS scale. Neurophysiological studies (somatosensory and motor evoked potentials, electromyography). Neuroimaging changes (magnetic resonance imaging). Urodynamic studies. Adverse effects.

Exclusion criteria:

- Meta-analysis and systematic reviews.
- Narrative reviews and expert opinions.
- MSC studies were performed exclusively on animals and in vitro.
- Non-mesenchymal stem cell studies in humans
- Non-traumatic spinal cord injuries.

### 2.3. Analyzed Variables

The following variables were analyzed:

- Sociodemographic variables: age, gender, level of injury, and extent of spinal cord injury according to the AIS scale.
- Study variables: author, year, type of study, randomization, control group, sample size, level of evidence, and methodological quality.
- Therapy-related variables: type of stem cells, route of administration, and dose administered.
- Efficacy outcome variables: improvement in AIS grade, improvement in ASIA sensory and motor scores, neurophysiological studies, urodynamic studies, neuroimaging studies, and adverse events.

In this systematic review, in order to avoid duplication of data, it was decided not to include previous meta-analyses or systematic reviews since most of the studies to be analyzed in this work were already included in them. Furthermore, it was decided to check for the possible retrieval of studies not found with the initial search strategy.

### 2.4. Quality Assessment

Once the search was conducted and potentially relevant studies were selected, the Mendeley Cite bibliographic citation manager was used to remove duplicate studies and manage bibliographic references. In addition, the references to these articles were reviewed to look for potentially useful studies not identified by the initial search strategy.

The methodological quality of the studies included in this review was assessed using the appropriate scale for the type of study. Clinical trials were assessed using the Physiotherapy Evidence Database PEDro scale, which has 11 items. This scale is divided into four categories: excellent (9–10 points), good (6–8 points), fair (4–5 points), and poor (<4 points) [67].

**Figure 3:**
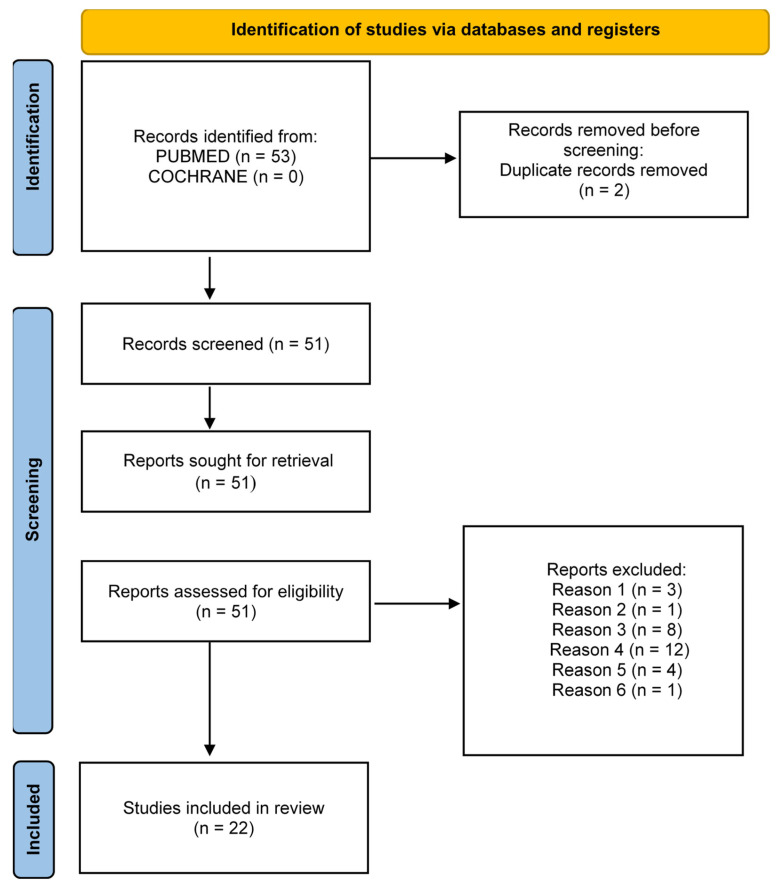
Flow chart representing the selection process followed to include studies in the review (modified from Page MJ et al.) [96].

This systematic review was conducted in accordance with the Preferred Reported Items for Systematic Reviews and Meta-Analyses (PRISMA) statement for the year 2020 [68]. Figure 3 shows the selection process for the articles included in this systematic review. Fifty-three studies were selected from the PUBMED and COCHRANE databases, of which two were excluded because they were duplicates. Of the 51 records screened, all publications were sought for retrieval. Of the publications assessed for eligibility, after applying the inclusion and exclusion criteria explained earlier in this section, 29 were excluded for the reasons shown in Table 3.

**Table 3:** Reasons why studies were excluded after applying the inclusion and exclusion criteria.

Reason 1	Studies by Moviglia G et al., Zamani H et al., Oraee-Yazdani S et al. used cell types other than MSCs [64,69,70]
Reason 2	The study by Xie Z et al. was published in Chinese [71]
Reason 3	The studies by Oliveri R et al., Jeon SR P et al., Deng L et al., Yang Z et al., Shang Z et al., Sarveazad A et al., Lu Y et al., Shang Z et al.; were conducted in animals [72,73,74,75,76,77,78,79]
Reason 4	Studies by Zhang D et al., Chen X et al., Khan S et al., Xu P et al., Yousefifard M et al., Muthu S et al., Chen W et al., Tang Q et al., Johnson L et al., Kvistad C et al., Xu X et al., Liu S et al. were meta-analyses or systematic reviews [11,80,81,82,83,84,85,86,87,88,89,90]
Reason 5	Studies by Xiao Z et al., Li Z et al., Zhao Y et al., Deng W et al. combined stem cells with other therapies [91,92,93,94]
Reason 6	Study by Vaquero J et al. included patients with syringomyelia [95].

Table 4 shows a list of the selected studies with the following data: type of study, journal of publication, presence or absence of a control group, and methodological quality according to the aforementioned scales.

## 3. Results and Discussion

Nowadays, TSCI is considered a virtually definitive event in which a series of pathophysiological mechanisms lead to the destruction of neuronal axons and irreversible spinal cord injury. The outcome is related to the degree and extent of the injury: complete injuries show very limited capacity for neurological recovery due to the limited ability of the CNS to regenerate cell and myelin loss and re-establish neuronal connections [38].

The search for an effective neuroprotective strategy to prevent secondary injury in an acute TSCI setting remains a priority in both clinical and basic research. Current treatments are based on attenuation of the secondary injury and include hemodynamic control and early surgical decompression. On the other hand, while recent studies have documented that rehabilitation can play a key role in spinal cord regeneration and plasticity, the goals of such therapy in complete spinal cord injury are limited because motor recovery is unlikely [33]. The most commonly used pharmacological agents for neuroprotection are corticosteroids, whose use has been abandoned due to a lack of evidence and numerous side effects [34]. Within neuroprotective strategies, numerous studies and clinical trials have investigated the efficacy of stem cells in the treatment of TSCI due to their ability to regulate the immune response, a function they perform through the production of growth factors such as hepatocyte growth factor, interleukin 10, or transforming growth factor beta 1 [7].

MSCs are currently one of the most prominent cell types in TSCI cell therapy research, not only because of their capacity for neuronal differentiation and regeneration but also because of their anti-apoptotic, anti-inflammatory, and angiogenic properties [58,59]. Their therapeutic properties, in addition to their progenitor capacity for chondrogenic, osteogenic, and adipogenic lineages, are based on the paracrine properties of some strains, such as umbilical cord MSCs, which involve the release of factors related to tissue repair [119]. Therefore, the aim of this systematic review was to analyze the safety, efficacy, and potential role of MSC therapies in the treatment of patients with traumatic spinal cord injuries.

### 3.1. Overview of Studies Included in the Systematic Review

Table 5 and Table 6 show the results of the 22 studies finally included in this systematic review, of which 21 were clinical trials and one was a case series, with a total of 463 patients. In all of these studies, there was a large predominance of males over females, representing 80.60% and 19.40% of the total, respectively. With regard to age, there was a large variability in the subjects included, with an age range between 16 and 66 years. These data are estimates, as three studies did not report the sex or age of the patients [101,104,108,109].

All studies included patients with chronic and subacute TSCI, except for Geffner L. et al. [98] and Honmou O. et al. [118], which included patients with acute SCI. The most commonly affected neurological level in patients was dorsal (49.02%), followed by cervical (41.48%), dorso-lumbar (5.40%), and lumbar (4.10%).

Of the 22 trials reviewed, 16 used bone marrow MSCs, 3 used adipose tissue MSCs, and 3 used umbilical cord MSCs as therapy, the latter being the studies by Cheng H et al. [104], Albu S et al. [116], and Yang Y et al. [118], which were the only ones to use allogeneic cells. Most of the studies in this review used bone marrow MSCs, probably because this was the first MSC type to be used; other possible reasons are the relatively high number of available studies on them and their easy extraction and processing.

In the majority of patients, the route of administration was intrathecal (14 trials), while it was intravenous in 2 trials and intralesional in 1 trial. Three other studies used intralesional and intrathecal routes together, Syková E. et al. [97] combined intrathecal and intravenous routes, and Geffner L. et al. [98] combined intrathecal, intravenous, and intralesional routes. While the cell dose administered to each patient was not reported in most of the studies, the dose used in the study with the highest number of patients was 2 × 10^6^ cells/kg [105].

The most common AIS grades in traumatic SCI patients were A (65.67%) and B (25.65%), with grades C and D being less common (7.86% and 0.82%, respectively). Four studies did not report the pre-treatment AIS grade of patients [100,101,102,118]. The higher frequencies of AIS grades A and B in most studies are probably due to the higher incidence of TSCI in these grades; on the other hand, the most severe grades are often initially used to test treatment safety in clinical trials in order to prevent adverse effects from significantly impacting the neurological function of patients. However, studies including more patients with incomplete motor impairment (AIS grades C and D) would be very informative for research since, as suggested by the interesting results of the studies included in this review, the potential benefits for this group of patients could have a very important impact from a functional point of view.

### 3.2. Outcomes following Cell Therapy

The following parameters were used to assess the efficacy of MSC therapy: improvement in AIS grade, improvement in sensory and/or motor scores on the ASIA scale, changes observed in imaging tests (magnetic resonance imaging), and changes in the results of neurophysiological and urodynamic studies. In addition, in order to assess the safety of the treatment, possible adverse effects following cell therapy administration were analyzed.

#### 3.2.1. AIS Grade

Of the 22 trials analyzed, only 4 reported no change in AIS grade, most notably the randomized trial by Albu S. et al. [116]. Five studies did not specify whether there was a change in AIS grade, and the study by Vaquero J. et al. [113] showed a significant improvement but did not specify the proportion. The remaining 12 studies showed improvements in AIS grades ranging from 7.69% in the study by Bhanot et al. to 100% in the study by Thakkar et al. [100,110]. Out of these twelve, Karamouzian S. et al., Dai G. et al., and El-Kheir W. et al. were control group studies and showed improvements of 31.26%, 45%, and 34%, respectively [102,103,105].

#### 3.2.2. ASIA Sensory Score

In 5 of the trials, the ASIA sensory score was not reported, while in 2 other trials, improvements were reported but were not significant [99,104]. In the remaining 15 studies, increases in the sensory score were reported, with the studies by Geffner L. et al. and Vaquero J. et al. showing the highest percentages of improvement, with 100% for both of them [98,111], followed by those by Mendonça M. et al. [106], Larocca et al. [112], and Hur J. et al. [107], with 57.14%, 60%, and 71.42%, respectively.

#### 3.2.3. ASIA Motor Score

In 3 of the trials, there were no improvements in ASIA motor scores, and 4 trials did not state whether there was an improvement or not. On the other hand, there was evidence of improvement in the studies by Pal R. et al. and Cheng H. et al., but they were not significant [99,104]. The remaining 14 trials showed significant improvements in patients’ motor function, but only 6 of these trials specified the degree of improvement, which ranged from 12.5% in the study by Oh S. et al. [108], with 16 patients, to 100% in the studies by Geffner et al. and Mendonça M. et al., with 8 and 14 patients, respectively [98,106].

#### 3.2.4. Neurophysiological Studies

Neurophysiological assessment included somatosensory evoked potentials, motor evoked potentials, and electromyography. Eleven studies did not monitor neurophysiological parameters; two showed no significant changes, and the remaining nine showed significant changes. Of these, 6 studies did not specify the type of improvement, while the studies by Mendonça M. et al. [106] and Hur J. et al. [107] reported changes in somatosensory evoked potentials, and Syková E. et al. reported improvements in both somatosensory and motor potentials in 7 out of 20 patients [97].

#### 3.2.5. Urodynamic Studies

The studies by Vaquero J. et al. were the only ones to specifically describe changes in urodynamic parameters, highlighting improvements in bladder capacity and compliance and detrusor pressure [111,113,114]. The studies by Dai G. et al. [103] and Yang Y. et al. [118] reported reductions in residual urine volume. Larocca T. et al. [112] and Pal R. et al. [99] reported improved sphincter control in 2 out of 5 and in 3 out of 30 patients, respectively, allowing for urinary catheter removal. On the other hand, the studies by Bhanot Y. et al. [100] and Thakkar et al. [110] reported improvements in bladder filling sensation in some patients. In 3 other trials, there were improvements in urodynamic studies, but their extent was not specified, and in the remaining 10 trials, no improvements were reported.

#### 3.2.6. Neuroimaging Studies (MRI Findings)

An MRI was used to assess radiological changes. In 36.36% (8 out of 22) of the trials, no imaging tests were performed, and in a further 36.36% (8 out of 22), no changes at the injury level were observed between images taken before and after cell therapy. In the studies where changes occurred, the most common were increased medullary diameter, decreased lesion cavity size, decreased lesion hyperintensity on T2 sequences, and gliosis.

#### 3.2.7. Severe Adverse Events

No trials reported serious treatment-related adverse events such as death, tumors, or immune reactions. Seven trials reported mild to moderate adverse events such as fever, headache, or neuropathic pain. Yang Y. et al. report 81 mild adverse events in the 41 patients included in the study [118]. The study by Vaquero J. et al. reports 69 adverse events in the 12 patients in the study. Of all the adverse events, 36.6% were related to the surgical procedure or cell therapy administration, with the most common ones being pain, fever, and seroma at the surgical site [111].

In this systematic review of the available scientific literature on MSC transplantation for TSCI, it was found that in controlled studies, patients who received MSC therapy improved their AIS by at least one grade, with most studies also showing improvement in sensory scores and, to a lesser extent, motor scores. On the other hand, only half of the studies used neurophysiological tests, and most of them did not specify the changes they produced. Similarly, only one study without a control group reported urodynamic changes.

Some studies used neuroimaging analyses to monitor changes in the area of the lesion after administering cell therapy, such as cavity size, the appearance of new areas of gliosis, or the formation of ectopic tissue [106,114]. In terms of neuroimaging evidence, findings such as decreased lesion cavity size, decreased lesion hyperintensity, and gliosis were observed in a few studies. Although these changes were reported in studies that found improvements in AIS grade, none of the studies analyzed in this review attempted to link these changes to recovery, and no definitive conclusions can therefore be drawn as to the influence that neuroimaging findings may have on recovery. In terms of safety, one third of the trials reported mild or moderate adverse effects related to the route of administration, and none reported serious treatment-related adverse effects.

Our results are consistent with the findings of the recent meta-analyses by Muthu S. et al. [10] and Chen W. et al. [85], studies with a large number of patients that were not included in this review due to the methodology used, as detailed above. With regard to the studies included in our review, most of them agreed on the benefits of MSC therapy, but the methodological quality according to the PEDro scale (applied to 21 of the 22 studies, as the study by Honmou O. et al. was a case series [117]) was poor or fair in 66% of the studies (14 out of 21), good in 28.6% (6 out of 21), and excellent only in the study by Albu S et al., which showed significant improvements in ASIA sensory scores only in the segments adjacent to the injury [66,116]. It is also worth noting that only five of the included studies were clinical trials with control groups. Nevertheless, it is noteworthy that all authors reported some benefits from this treatment and agreed that this treatment strategy is one of the most interesting lines to pursue in order to achieve recovery in TSCI.

Among the most interesting points to be discussed in MSC treatment are the cell lines to use, the route and dose of administration, the type of TSCI, and the appropriate stage of development for transplantation.

Although MSC sources are widely available, the ideal stem cell type remains to be clarified, as the exact type of stem cell has not been determined due to a lack of experience in clinical practice. Thus, autologous bone marrow MSCs prevent autoimmune reactions, adipose tissue MSCs are widely available and easily accessible, and umbilical cord MSCs require a complex preparation process to prevent immunological reactions [11]. In addition, although their properties and, to some extent, their mechanisms of action are known, the changes they cause in injured tissues and how they can be more specifically targeted by neuroimaging or electrophysiological testing remain to be determined.

In terms of route of administration, cells can be transplanted intrathecally, intralesionally, intravenously, and even intra-arterially. This aspect is another important parameter to take into account in therapy, as it has been shown that the use of one route or the other can influence efficacy, as is the case in the study by Syková E. et al., which, using the intra-arterial and intravenous routes, found that the group of patients who received intra-arterial therapy showed no benefits [97].

Currently, the most commonly used route is the intrathecal route, which appears to produce better results than the intravenous and intralesional routes in terms of ASIA motor and sensory scores and the incidence of adverse effects, as demonstrated by most of the studies in this review [85]. Another aspect that could influence the efficacy of the therapy, related to the route of administration, is the cell dose to be infused, as the optimal dose has not been clearly determined so far; for example, Vaquero J et al. obtained different results in their studies using doses between 30 × 10^6^ and 100 × 10^6^ cells in different intrathecal infusions [111,113,114].

Another important consideration in terms of potential benefits, not only regenerative but also functional, is the degree and stage of development of the TSCI. In this sense, virtually all the patients included in this review were patients in the subacute and chronic phases and with complete motor spinal cord injury (AIS grades A and B). Therefore, given the anti-apoptotic and anti-inflammatory functions of MSCs, it would be very interesting to evaluate these possible effects in patients in the acute phase of injury to observe their potential clinical benefits. Similarly, studies including more patients with incomplete motor impairment (AIS grades C and D) could be very informative for research, as the possible benefits for this group of patients could be very important from a functional point of view, as can be deduced from the interesting results of the studies included in this review.

Assessment of outcomes after treatment, as seen in most of the studies in this review, is based on clinical evidence, the interpretation of which may be subject to bias. Therefore, when studies report a significant change in ASIA sensory or motor scores, it is worth asking ourselves whether it has any real value or is simply a statistical parameter that, even if significant, may be explained by variability. Thus, some authors, such as Evaniew et al., determined in an observational study that the variability in the time of the neurological examination within hours after an acute traumatic SCI may influence the observations of long-term neurological recovery [120]. In addition, Burns et al. evaluated the reliability of the early ISNCSCI examination (performed < or = 48 h post-injury) in complete motor SCI. These authors identified multiple factors, including mechanical ventilation, intoxication, concomitant blunt head injury, psychological disorders, and severe pain, that could potentially affect the reliability of the baseline examination [121]. On the other hand, as it is a clinical examination, we know that the differences in the results can be justified by the variability between the observers or even between different examinations carried out by the same observer. On the other hand, not all trials use the same methods to assess patients, and it is therefore necessary to standardize the tools and their parameters in order to assess the possible benefits of this treatment in a more objective and homogeneous way.

Although the promising potential results of Mesenchymal Stem Cell Therapy are highlighted in this review, there are also other future therapies in development. Thus, according to a recent review by Cunningham CJ et al., gene therapies show promise in preclinical models of SCI, although there is no consensus on which genes are more effective in promoting axonal regeneration [122]. Nanomaterials are also opening new avenues in SCI treatment. Its possible mechanisms of action are the direct protection and maintenance of nerve cells, protecting nerve cells by improving the damaged microenvironment during the process of secondary injury, or facilitating neural regeneration [123]. Neurostimulation combined with rehabilitation has recently been applied to patients with complete and incomplete SCI, with promising results. Courtine G. et al. were able to restore gait in people with SCI through epidural electrical stimulation targeting the dorsal roots of lumbosacral segments [124,125].

This review has had some limitations in its implementation. Firstly, the search for studies was not easy, as this is a very recently developed topic about which the available studies are scarce, difficult to find, and often written in languages other than English and Spanish. Secondly, most of the studies do not have a control group, are not randomized, are very heterogeneous, show low methodological quality, and do not provide very detailed information about how the whole process was carried out or how long the patients were followed up. Moreover, three out of 22 studies did not report the age or sex of the patients.

Thirdly, the number of patients available and included in this review was limited. There are several reasons for this: TSCI is a rare condition among the population that is constantly being researched and where new strategies and lines of treatment are gradually being developed. MSC treatment is a new line of research with little scientific evidence to date; therefore, the data in this review should be interpreted with caution, as in some cases the efficacy and potential benefits of MSC use in TSCI have been overestimated. In view of all this, it is important to emphasize the need for multi-center, randomized, and controlled trials with large numbers of patients and over a long period of time, which will allow us to draw firm conclusions.

## 4. Conclusions

Mesenchymal stem cell therapy leads to neurological improvement in traumatic spinal cord injury patients. Positive changes in AIS grade and in ASIA sensory and motor scores were observed in patients. In addition, the potential safety of this therapy in the short and medium term was demonstrated.

The clinical and functional significance of these advances, as well as their long-term safety, remain to be determined, as there is little scientific evidence and no general recommendation for their use.

From this review, it can be concluded that stem cell therapy is considered the most promising treatment for traumatic spinal cord injury and the one on which all efforts are focused. For this reason, more multicenter, randomized, and controlled trials with larger numbers of patients are needed, together with the standardization of complementary assessment tests and their parameters, such as new injury biomarkers, in order to evaluate the possible benefits of this treatment in a more objective and homogeneous way, thus allowing us to draw definitive conclusions. However, there are factors that remain significant barriers for multicenter, randomized, and controlled trials with larger numbers of patients, such as low patient recruitment due to the low incidence of TSCI, stem cell production, and financing, among others.

## Figures and Tables

**Table 1:** Warning signs and symptoms of spinal cord injury (modified from Galeiras Vázquez R. et al.) [5].

Warning Signs and Symptoms of Spinal Cord Injury
-Limb weakness or paralysis
-Trunk or limb sensory disorders
-Difficulties with language skills (hypophonia)
-Abdominal breathing
-Hypotension and paradoxical bradycardia
-Elbow flexion position
-Spinal cord pain or deformity
-Paresthesias or electric shock feeling
-Absence of pain when predictably painful lesions exist
-Priapism

**Table 2:** ASIA Impairment Scale.

A = Complete. no sensory or motor function is preserved in the sacral segments S4–S5
B = Sensory incomplete. Sensory but not motor function is preserved below the neurological level and includes the sacral segments S4-S5 (light touch or pin-prick at S4–S5 or deep anal pressure) AND no motor function is preserved more than three levels below the motor level on either side of the body
C = Motor incomplete. Motor function is preserved below the neurological level AND more than half of the key muscle functions below the neurological level of injury have a muscle grade less than 3 (grades 0–2)
D = Motor incomplete. Motor function is preserved below the neurological level AND at least half (half or more) of the key muscle functions below the neurological level of injury have a muscle grade ≥3
E = Normal. If sensation and motor function as tested with the ISNCSCI are graded as normal in all segments AND the patient has prior deficits, then the AIS grade is E. Someone without an initial SCI does not receive an AIS grade

**Table 4:** Registry of the studies included in the systematic review.

Article	Journal	Type of Study	Randomi-Sation	Control Group	Methodological Quality (PEDro Scale)
Syková E et al. [97], 2006	Cell Transplantion	Clinical trial	NO	NO	3
Geffner L et al. [98], 2008	Cell Transplantion	Clinical trial	NO	NO	4
Pal R et al. [99], 2009	Cytotherapy	Clinical trial	NO	NO	3
Bhanot Y et al. [100], 2011	British Journal of Neurosurgery	Clinical trial	NO	NO	4
Ra J et al. [101], 2011	Stem Cells and Development	Clinical trial	NO	NO	3
Karamouzian S et al. [102], 2012	Clinical Neurology and Neurosurgery	Clinical trial	NO	YES	3
Dai G et al. [103], 2013	Brain Research	Clinical trial	YES	YES	5
Cheng H et al. [104], 2014	Journal of Translational Medicine	Clinical trial	YES	YES	8
El-Kheir W et al. [105], 2014	Cell Transplantion	Clinical trial	YES	YES	7
Mendonça M et al. [106], 2014	Stem Cell Research and Therapy	Clinical trial	NO	NO	6
Hur J et al. [107], 2016	Journal of Spinal Cord Medicine	Clinical trial	NO	NO	4
Oh S et al. [108], 2016	Neurosurgery	Clinical trial	NO	NO	6
Satti H et al. [109], 2016	Cytotherapy	Clinical trial	NO	NO	4
Thakkar U et al. [110], 2016	Advanced Biomedical Research	Clinical trial	NO	NO	4
Vaquero J et al. [111], 2016	Cytotherapy	Clinical trial	NO	NO	5
Larocca T et al. [112], 2017	Cytotherapy	Clinical trial	NO	NO	4
Vaquero J et al. [113], 2017	Cytotherapy	Clinical trial	NO	NO	4
Vaquero J et al. [114], 2018	Cytotherapy	Clinical trial	NO	NO	5
Yang Yalin Z et al. [115], 2020	International Journal of Clinical and Experimental Medicine	Clinical trial	YES	YES	8
Albu S et al. [116], 2021	Cytotherapy	Clinical trial	YES	NO	9
Honmou O et al. [117], 2021	Clinical Neurology and Neurosurgery	Case Series	NO	NO	
Yang Y et al. [118], 2021	Cytotherapy	Clinical trial	NO	NO	6

**Table 5:** Characteristics of the articles included in the systematic review.

Article	Age	Gender		Sample Size	Type of TSCI	Injury Level	Type of Mesenchymal Stem Cell	Route of Administration
		Men	Women					
Syková E et al. [97], 2006	19–41	16	4	20	13 Chronic 7 Subacute	12 Cervical 8 Thoracic	Autologous bone marrow	Intra-arterial Intravenous
Geffner L et al. [98], 2008	28–44	7	1	8	4 Chronic 4 Acute	Thoracic	Autologous bone marrow	Intralesional Intrathecal Intravenous
Pal R et al. [99], 2009	17–56	27	3	30	10 Subacute Chronic	7 Cervical 23 Thoracic	Autologous bone marrow	Intrathecal
Bhanot Y et al. [100] 2011	18–52	10	3	13	Chronic	5 Cervical 8 Thoracic	Autologous bone marrow	Intralesional Intrathecal
Ra J et al. [101], 2011	N.D.	8	0	8	Chronic	Unspecified	Autologous adipose tissue	Intravenous
Karamouzian S et al. [102], 2012	23–48	24	7	31	Subacute	Thoracic	Autologous bone marrow	Intrathecal
Dai G et al. [103], 2013	22–54	28	12	40	Chronic	40 Cervical	Autologous bone marrow	Intrathecal
Cheng H et al. [104], 2014	27–43	Unspecified		10	Chronic	10 Dorsal-lumbar	Allogeneic umbilical cord cells	Intralesional
El-Kheir W et al. [105], 2014	16–45	61	9	70	Chronic	53 Thoracic 17 Cervical	Autologous bone marrow	Intrathecal
Mendonça M et al. [106], 2014	18–65	10	4	14	Chronic	14 Dorsal-lumbar	Autologous bone marrow	Intrathecal
Hur J et al. [107], 2016	20–66	12	2	14	10 Chronic 4 Subacute	6 Cervical 7 Thoracic 1 Lumbar	Autologous adipose tissue	Intrathecal
Oh S et al. [108], 2016	18–65	Unspecified		16	Chronic	Cervical	Autologous bone marrow	Intralesional Intrathecal
Satti H et al. [109], 2016	24–38	Unspecified		9	6 Chronic 3 Subacute	Thoracic	Autologous bone marrow	Intrathecal
Thakkar U et al. [110], 2016	9–42	8	2	10	Chronic	6 Dorsal 3 Dorsal-lumbar 1 Lumbar	Autologous adipose tissue	Intrathecal
Vaquero J et al. [111], 2016	32–50	9	3	12	Chronic	Thoracic	Autologous bone marrow	Intralesional Intrathecal
Larocca T et al. [112], 2017	36–52	5	0	5	Chronic	Thoracic	Autologous bone marrow	Intrathecal
Vaquero J et al. [113], 2017	33–51	8	2	10	Chronic	5 Cervical 2 Thoracic 3 Lumbar	Autologous bone marrow	Intrathecal
Vaquero J et al. [114], 2018	28–62	7	4	11	Chronic	4 Cervical 4 Thoracic 3 Dorsal-lumbar	Autologous bone marrow	Intrathecal
Yang Yalin et al. [115], 2020	27–43	53	15	68	Chronic	44 Cervical 24 Thoracic	Autologous bone marrow	Intrathecal
Albu S et al. [116], 2021	25–47	7	3	10	Chronic	Thoracic	Allogeneic umbilical cord cells	Intrathecal
Honmou O et al. [117], 2021	21–66	12	1	13	Acute	Cervical	Autologous bone marrow	Intravenous
Yang Y et al. [118], 2021	18–65	33	8	41	Chronic	24 Cervical 7 Thoracic 10 Dorsal-lumbar	Allogeneic umbilical cord cells	Intrathecal

**Table 6:** Results of the articles included in the systematic review.

	AIS * Grade				AIS Grade Improvement		ASIA ** Sensory Score Improvement		ASIA Motor Score Improvement		Image Test (MRI)	Improvement in Neurophysiological Studies	Improvement in Urodynamic Studies	Serious Adverse Effects
	A	B	C	D	Yes	No	Yes	No	Yes	No				
Syková E et al. [97], 2006	15	4	1	0	3	17	6	14	6	14	Gliosis	4 Improve in SSEPs *** 3 Improve in MEPs ****	Not done	No
Geffner L et al. [98], 2008	5	1	2	0	6	2	8	0	8	0	Reduction of cavity and lesion hyperintensity	Not done	Yes	No
Pal R et al. [99], 2009	24	0	6	0	No improvement		Non-significant improvement		Non-significant improvement		No significant changes	No significant changes	3 Improved sphincter control	No
Bhanot Y et al. [100], 2011	Unspecified				1	12	3	9	No improvement		Not done	Not done	1 Improved bladder fullness sensation	Mild side effects
Ra J et al. [101], 2011	Unspecified				Unspecified		Unspecified		Unspecified		No significant changes	Not done	Not done	No
Karamouzian S et al. [102], 2012	Unspecified				5	11	Significant improvement		Significant improvement		Not done	Not done	Not done	No
Dai G et al. [103], 2013	40	0	0	0	9	11	Significant improvement		Significant improvement		No significant changes	Yes	Reduction in residual urine volume	No
Cheng H et al. [104], 2014	10	0	0	0	Unspecified		Non-significant improvement		Non-significant improvement		Not done	Not done	Yes	No
El-Kheir W et al. [105], 2014	25	45	0	0	17	33	Significant improvement		Significant improvement		Gliosis and reduced lesion hyperintensity	Yes	Not done	No
Mendonça M et al. [106], 2014	14	0	0	0	7	7	8	6	14	0	No significant changes	Improve in SSEPs	Yes	No
Oh S et al. [107], 2016	1	15	0	0	No improvement		Unspecified		2	14	5 Increase in medullary diameter 1 Disappearance of cavity 3 Reduction of cavity	Yes	Unspecified	8 Mild adverse events
Satti H et al. [108], 2016	9	0	0	0	Unspecified		Unspecified		Unspecified		No significant changes	Not done	Unspecified	3 Mild adverse events
Hur J et al. [109], 2016	12	1	0	1	No improvement		10	4	5	9	No significant changes	Minimum SSEPs change	Unspecified	4 Mild adverse events
Thakkar et al. [110], 2016	10	0	0	0	10	0	Unspecified		Unspecified		Not done	Not done	5 Improved bladder fullness sensation	Mild adverse events
Vaquero J et al. [111], 2016	12	0	0	0	4	8	12	0	9	3	5 Reduction or disappearance of lesion hyperintensity	Yes	Improvement in bladder capacity and compliance and in the detrusor muscle pressure	69 Mild to moderate adverse events (22 related to surgery)
Larocca T et al. [112], 2017	5	0	0	0	1	4	3	2	No improvement		Not done	Not done	2 Improved sphincter control, 1 Bladder filling sensation	No
Vaquero J et al. [113], 2017	0	4	5	1	Significant improvement		Significant improvement		Significant improvement		No significant changes	Yes	Improvement in bladder capacity and compliance and in the detrusor muscle pressure	Acute non-treatment-related bronchitis
Vaquero J et al. [114], 2018	3	4	3	1	3	8	Significant improvement		Significant improvement		No significant changes	Yes	Improvement in bladder capacity and compliance and in the detrusor muscle pressure	No
Yang Yalin et al. [115], 2020	42	16	10	0	Unspecified		Significant improvement		Significant improvement		Not done	Not done	Not done	64 Mild adverse events
Albu S et al. [116], 2021	10	0	0	0	No improvement		Significant improvement		No improvement		Not done	No significant changes	No significant changes	No
Honmou O et al. [117], 2021	6	5	2	0	12	1	Unspecified		Unspecified		Not done	Not done	Unspecified	No
Yang Y et al. [118], 2021	Unspecified				Unspecified		Significant improvement		Significant improvement		Unspecified	Not done	Reduction in residual urine volume	81 Mild adverse events

* AIS: ASIA Impairment Scale. ** ASIA: American Spinal Injury Association. *** SSEPs: Somatosensory Evoked Potentials. **** MEPs: Motor Evoked Potentials.

## Data Availability

Not applicable.
